# Cervicomediastinal Hematoma From Inferior Thyroid Artery Injury After Attempted Permanent Dialysis Catheter Placement Without Ultrasound Guidance

**DOI:** 10.7759/cureus.91889

**Published:** 2025-09-09

**Authors:** Grażyna Waśka, Pawel Radkowski, Hubert Oniszczuk, Piotr Nowakowski, Elwira Misztela-Lisiecka

**Affiliations:** 1 Department of Internal Medicine, Specialist Hospital No. 1 in Bytom, Bytom, POL; 2 Department of Anaesthesiology and Intensive Care, Collegium Medicum, University of Warmia and Mazury in Olsztyn, Olsztyn, POL; 3 Department of Anaesthesiology and Intensive Care, Medical University of Białystok, Białystok, POL; 4 Department of Internal Medicine, Municipal Hospital in Gliwice, Gliwice, POL; 5 Department of Internal Medicine, Pabianice Medical Centre, Pabianice, POL

**Keywords:** central venous catheterization, inferior thyroid artery injury, mediastinal hematoma, permanent dialysis catheter, transcatheter arterial embolization

## Abstract

Permanent dialysis catheter (PDC) placement is a routine hospital procedure, but it may lead to rare and life-threatening complications. We present the case of a 41-year-old woman who developed hemomediastinum caused by injury to a branch of the inferior thyroid artery during internal jugular vein cannulation using anatomical landmarks. The patient developed neck swelling and respiratory symptoms shortly after the procedure. Diagnosis was confirmed via imaging, and successful hemostasis was achieved with transcatheter arterial embolization. This case highlights the importance of careful technique and prompt recognition of rare vascular injuries.

## Introduction

Hemodialysis (HD) remains one of the most widely used renal replacement therapies worldwide, alongside peritoneal dialysis and kidney transplantation. Over two million patients currently receive HD globally. Effective HD requires reliable vascular access, which may be achieved via an arteriovenous fistula (AVF), vascular graft, or permanent dialysis catheter (PDC). While AVF is generally preferred due to its lower complication rates, it requires several weeks to mature and may not be feasible in patients with heart failure, chronic lung disease, or steal syndrome. In such cases, a temporary or PDC becomes necessary [1].

Despite its clinical utility, PDC placement is associated with several complications, including pneumothorax, arterial puncture, hematoma, infection, thrombosis, and central venous stenosis. Traditional insertion techniques rely on anatomical landmarks, which result in lower success rates (60-90%) and higher complication risks (5-20%). In contrast, the use of real-time ultrasound (US) guidance significantly reduces these risks, increases first-attempt success rates, and enhances the overall safety of the procedure [2].

Although rare, injury to small cervical arteries such as the inferior thyroid artery (ITA) during internal jugular vein cannulation has been reported and may result in life-threatening bleeding. Due to the anatomical location and vascular supply of the neck and upper mediastinum, such injuries may result in rapidly expanding hematomas with airway compromise. In these cases, prompt recognition and management are essential, as delays may lead to fatal outcomes. Treatment strategies include surgical ligation or, increasingly, transcatheter arterial embolization (TAE), which is considered an effective and minimally invasive approach [3].

Hemorrhagic complications involving atypical vessels, such as those supplying the mediastinum or lower neck, are sparsely reported in the literature. However, awareness of these rare bleeding sources is critical for accurate diagnosis and timely intervention. TAE has gained recognition as the first-line treatment in such presentations, offering a safe and efficient therapeutic option in select patients [4].

In this report, we present the case of a patient who developed a life-threatening cervical hematoma with impending airway obstruction due to iatrogenic arterial injury during attempted PDC placement, successfully treated with endovascular embolization.

## Case presentation

**Figure 1:**
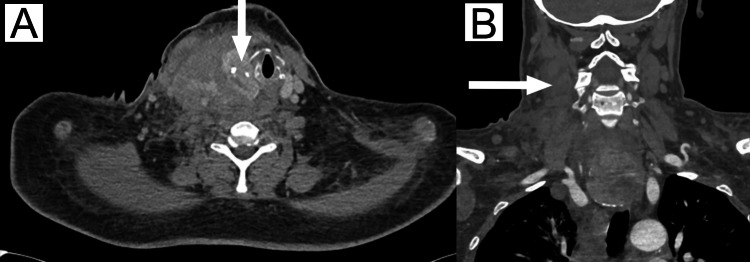
Radiological imaging (A) Axial contrast-enhanced CT image of the neck demonstrating a large right-sided cervical hematoma (arrow) causing marked leftward displacement of the trachea. (B) Coronal reformatted CT image showing the full craniocaudal extent of the hematoma (arrow) extending into the superior mediastinum.

A 41-year-old woman with end-stage renal disease on HD, hypertension, obesity, anemia, and a newly diagnosed left breast malignancy under investigation, without clinical or radiological evidence of goiter, was urgently transferred to the intensive care unit (ICU) from the Department of Vascular Surgery in June 2025. She had initially been transported by helicopter emergency services from a dialysis unit due to active bleeding from the region of the thyroid tumor capsule, following an attempted PDC placement into the right internal jugular vein. The catheter was approximately 20 cm in length, and US guidance was not available at the time. Emergency imaging revealed a right-sided cervical hematoma and suspected active bleeding from the ITA (Figures 1A, 1B). The patient was intubated, sedated, and referred for endovascular embolization of the bleeding site. Before intubation, the patient experienced increasing dyspnea and inspiratory stridor suggestive of progressive airway obstruction, and she was unable to tolerate lying flat. She was then admitted to the ICU for further management.

Three weeks earlier, she had been hospitalized for *Staphylococcus aureus* sepsis and treated with meropenem, cloxacillin, and gentamicin. Blood cultures later revealed resistance to cloxacillin, leading to discontinuation of this antibiotic regimen. Further cultures were obtained, and vancomycin was considered pending results.

On ICU admission, the patient was sedated and ventilated with a fraction of inspired oxygen (FiO₂) of 70%, receiving continuous renal replacement therapy (CRRT) via a right femoral vein catheter, which was successfully recanalized and remained functional throughout her ICU stay. Analgesic sedation with propofol and oxycodone was maintained, and norepinephrine was administered at a low dose for hemodynamic support. Laboratory tests revealed anemia (Table 1), prompting transfusion of packed red blood cells and plasma. Despite the severity of her condition, inflammatory markers remained low, and she was afebrile. Over the following days, sedation was tapered, respiratory support was gradually reduced, and neurological responsiveness improved. The patient remained intubated for four days before successful extubation.

**Table 1:** Results of the complete blood count and arterial blood gas analysis Hemoglobin (g/dL: grams per deciliter); platelets (count/µL: count per microliter); pH (potential of hydrogen, measure of hydrogen ion concentration); PaCO₂ (partial pressure of carbon dioxide, mmHg: millimeters of mercury).

Parameters	Admission day	Day 1	Day 2	Day 3	Day 4	Day 5	Day 6	Day 8-10	Normal range
Hemoglobin, g/dL	7.9	7.2	7.5	8.7	8.1	7.7	8.1	9.4	12-16
Platelets, count/uL	216 × 10^3^	249 × 10^3^	174 × 10^3^	139 × 10^3^	136 × 10^3^	157 × 10^3^	180 × 10^3^	198 × 10^3^	150-400 × 10^3^
Arterial blood gases									
pH	7,302	7.363	7.424	7.426	7.423	7.466	7.437	7.435	7.35 - 7.45
PaCO2 (mmHG)	44.3	44.2	44.8	42.8	43.5	38.4	35.4	31.0	32.0-48.0

A contrast-enhanced computed tomography (CT) scan of the neck and chest was performed to assess the extent of the hematoma and its effect on airway patency. The follow-up CT scan revealed no signs of active bleeding in the neck, with partial reduction in the size of the cervical and upper mediastinal hematoma compared to the previous study performed shortly after the incident, raising suspicion of pneumonia.

After four days, the patient was successfully extubated. She was alert, able to follow commands, and maintaining spontaneous breathing with supplemental oxygen. Episodes of delirium were noted, requiring treatment with a dexmedetomidine infusion, hydroxyzine, and quetiapine. Although her neck remained swollen, airway patency was preserved, and there were no signs of stridor or respiratory distress. Renal replacement therapy continued with stable fluid balance and a gradual decrease in serum urea and creatinine. Broad-spectrum antibiotic therapy with meropenem was maintained.

## Discussion

PDCs remain a widely used method of vascular access for HD, particularly when urgent dialysis is indicated or when alternative options are not feasible. While AVFs are the preferred access due to their superior long-term outcomes, they require time to mature, often at least six weeks, and may not be suitable for patients with comorbidities such as heart failure, chronic lung disease, or steal syndrome. In contrast, PDC can provide immediate access, making it a practical solution in urgent settings or when AVF maturation is delayed or impossible [1].

The increasing use of PDCs can also be attributed to their relatively simple and quick insertion, painless connection to the dialysis circuit, and absence of significant hemodynamic load on the heart. However, these benefits come at the cost of a significantly higher complication rate compared to AVFs or vascular grafts. Complications include mechanical dysfunction, thrombosis, and, most notably, infections such as bacteremia. These infections contribute to increased morbidity and mortality in dialysis patients and represent the second leading cause of death in this population. Furthermore, PDC-related complications result in a substantial healthcare burden, with up to 1300 hospitalizations per 1000 patient-years reported due to infection alone [5]. 

To better understand the risks and appropriate use of PDCs, it is important to distinguish between the two main types of central venous catheters used for HD: nontunneled and tunneled. Nontunneled catheters are typically used in critically ill patients for short-term dialysis and are usually removed before hospital discharge due to high risks of infection and dislodgement. In contrast, tunneled catheters are suited for long-term dialysis, most often placed in the internal jugular vein and tunneled subcutaneously to reduce the risk of infection and enhance catheter stability [6].

This case highlights a rare but serious complication of arterial injury and subsequent cervicomediastinal hematoma following PDC placement without US guidance. US guidance is now the standard for internal jugular vein catheterization due to its ability to significantly reduce complications such as arterial puncture, hematoma, pneumothorax, and failure of catheter placement [7,8]. However, in emergent situations or where US is unavailable, clinicians rely on anatomical landmarks. This technique, although critical in emergencies, is less precise and linked with higher complication rates, especially in patients with difficult anatomy like obesity [2,8]. The lack of US guidance in this case likely contributed to the inadvertent arterial puncture and vessel injury.

While injuries to major arteries such as the carotid artery are well-documented, damage to smaller vessels like the ITA is rare but can lead to serious complications. Such injuries may cause expanding hematomas in the neck and upper mediastinum, potentially threatening airway patency and hemodynamic stability [3,4]. Awareness of these uncommon bleeding sources is important in patients presenting with neck swelling or respiratory distress after catheter insertion.

The patient developed thrombocytopenia after the complication, though baseline platelet counts prior to the procedure are unknown. Severe coagulopathy (platelet count less than 20 ×10⁹/L or international normalized ratio (INR) more than 3.0) increases bleeding risk during invasive procedures. Correction of coagulation abnormalities is recommended before catheter insertion, particularly when landmark techniques are used or the subclavian vein site is chosen, due to limited ability to control bleeding by compression [8].

Despite advances such as the Seldinger technique and widespread US use, complications persist, underscoring the importance of operator training, risk assessment, and US guidance to improve patient safety [7,8].

## Conclusions

PDC placement remains a vital procedure in modern medicine but carries risks that can lead to serious complications. This case highlights a rare but potentially life-threatening arterial injury resulting in a cervicomediastinal hematoma after PDC placement without US guidance. Prompt recognition, imaging, and multidisciplinary management are crucial for favorable outcomes. US guidance should be considered the standard of care to minimize such complications, especially in patients with challenging anatomy or coagulopathy. Awareness of rare vascular injuries and adherence to strict procedural protocols can improve patient safety and reduce morbidity associated with central venous access.
